# EEG beta-modulations reflect age-specific motor resource allocation during dual-task walking

**DOI:** 10.1038/s41598-021-94874-2

**Published:** 2021-08-09

**Authors:** Janna Protzak, Klaus Gramann

**Affiliations:** 1 Junior research group FANS (Pedestrian Assistance System for Older Road User), Technische Universitaet Berlin, 10587 Berlin, Germany; 2 Biological Psychology and Neuroergonomics, Technische Universitaet Berlin, 10623 Berlin, Germany

**Keywords:** Cognitive ageing, Cognitive neuroscience, Sensorimotor processing, Motor control

## Abstract

The parallel execution of two motor tasks can lead to performance decrements in either one or both of the tasks. Age-related declines can further magnify the underlying competition for cognitive resources. However, little is known about the neural dynamics underlying motor resource allocation during dual-task walking. To better understand motor resource conflicts, this study investigated sensorimotor brain rhythms in younger and older adults using a dual-task protocol. Time-frequency data from two independent component motor clusters were extracted from electroencephalography data during sitting and walking with an additional task requiring manual responses. Button press-related desynchronization in the alpha and beta frequency range were analyzed for the impact of age (< 35 years, ≥ 70 years) and motor task (sitting, walking). Button press-related desynchronization in the beta band was more pronounced for older participants and both age groups demonstrated less pronounced desynchronizations in both frequency bands during walking compared to sitting. Older participants revealed less power modulations between sitting and walking, and less pronounced changes in beta and alpha suppression were associated with greater slowing in walking speed. Our results indicate age-specific allocations strategies during dual-task walking as well as interdependencies of concurrently performed motor tasks reflected in modulations of sensorimotor rhythms.

## Introduction

During our daily routines we are constantly involved in the parallel execution of concurrent tasks like searching for the key in our pockets while walking. Especially in older adults, age-related declines in the sensory, motor, and cognitive domains can lead to resource conflicts and changes in resource allocation strategies in dual-task scenarios^1^. In a recent study, we demonstrated that performance decrements in a simple visual detection task during walking are reflected in attenuated early visual processing^2^. The results indicated cognitive-motor interference (CMI) during dual-task walking with an exceeding demand for attentional resources in the visual domain. In the present analysis, we focused on potential age-differences in resource allocation between two concurrently performed motor tasks - pressing a button in response to visually presented stimuli while walking.

The cortical engagement in motor tasks like button-presses can be evaluated with event-related time-frequency (TF) analyses of the electroencephalogram (EEG)^3,4^. Voluntary and imagined movements are characterized by changes in oscillatory activity in sensorimotor areas in the alpha (approximately between 8 and 12 Hz) and the beta frequency range (approximately between 16 and 30 Hz). Specifically, around the time of motor actions like button presses, decreased alpha and beta synchronization in sensorimotor areas relative to a baseline can be observed^5^.

In older compared to younger adults, such movement-related suppression, especially in beta sensorimotor rhythms, have been frequently reported to be more pronounced^6–8^. For manual button presses, Bardouille et al. (2019)^7^ showed in an analysis of a large data set (N > 500), that increasing age was associated with increasing beta suppression in primary motor cortex and somatosensory cortices. However, a comprehensive understanding and functional interpretation of local upregulation in beta suppression in older adults during button presses as compensatory or inefficient is not yet existing.

It further remains an open question whether age-related differences in alpha and beta sensorimotor rhythms during manual responses reflect adaptation in dual-task walking scenarios. Bradford et al. (2919)^9^ reported first results from a Mobile Brain/Body Imaging (MoBI)^10–12^ study with younger adults that demonstrated less pronounced desynchronization in the alpha and beta band in sensorimotor regions for manual responses to an oddball task during walking as compared to sitting. This result is generally in line with other MoBI walking studies that reported secondary task-related power decrements in mobile as compared to stationary conditions^13–15^. A common general interpretation for decreased task-related power during dual-task walking is that cognitive resources are drawn away from the parallel task to accomplish the walking task. However, direct associations between changes in behavior and neurophysiological measures are needed to assume such reductions as indicating dual-task costs and resource allocation mechanisms.

For the assessment of modulations in brain dynamics during motor dual-tasks, the combination of age group and motor task contrasts (sitting vs. walking) is well suited. Increasing age is hypothesized to be associated with increased button press-related desynchronization in the beta band, while walking is assumed to result in less pronounced desynchronization in the alpha and beta frequency bands. If increased button-press related desynchronization in older participants is reflecting compensatory resource allocation mechanism as a result of developmental changes, the oscillatory pattern underlying successful button presses should not be modulated during walking. Otherwise, dedifferent local upregulation in older participants during sitting can be assumed. Furthermore, age-related differences in resource allocation strategies can be assessed through contrasts of behavioural and neurophysiological results. To this end, we analyzed absolute values of each measure as well as dual-task costs in terms of relative changes between both motor task conditions.

## Method

The original study protocol was described in detail in a previous report^2^. The same data sets were used for the present analysis. However, we re-report essential study information here for reasons of clarity and consistency. For an extensive study description, we redirect the reader to the initial report.

### Participant sample

Data sets from 15 younger participants (7 female, mean age: 27 years, SD: 3.1 years, range: 19–31 years) and 15 older participants (9 female, mean age: 74 years, SD: 2.9 years, range: 70–80 years) were included in the analysis. All participants were right-handed (assessed by the Edinburgh Handedness Inventory, German adaption^16^), passed a cognitive screening (Montreal Cognitive Assessment, German adaptation^17^) as well as a peripheral visual perception test (Schuhfried GmbH, Mödling, Austria) and reported to be free of neurological diseases. Only participants with an active field of view of 120$^\circ$ were invited for participation. The study was approved by the local Ethics Committee of the Institute of Psychology and Ergonomics, TU Berlin and carried out in accordance with relevant guidelines and regulations set by the TU Berlin. Written informed consent was given by all participants.

### Experimental task and setup

Participants were asked to respond to brief (50ms) visual stimuli (yellow LED light) that were randomly presented in different angels (20$^\circ$, 40$^\circ$, 60$^\circ$) in either the left or the right peripheral visual field. Responses were executed via button presses on Bluetooth gaming controllers with the left and right index fingers congruent to the stimulus presentation side (left index finger presses for visual stimuli in the left visual field and right index finger presses for stimuli in the right visual field). The visual stimulus-response task was executed during three different motor tasks - sitting, standing, and walking. In the current analysis, we focus the statistical comparison on the sitting and walking condition, as these were identified as the conditions revealing the strongest effects in the previous analyses^2^. During sitting, the visual stimulation was delivered via LEDs mounted at fixed location with participants’ head position fixed with the help of a chin rest. In the walking condition, participants walked up and down between two LED arrays of ten meter length. Stimulus presentation positions corresponding to the desired presentation angles were determined based on the actual head position. To this end, the head position was calculated continuously via optical motion tracking data (Impulse X2 System, PhaseSpace Inc., San Leandro, CA, USA). The momentary head orientation was used to compute the eccentricity for stimulus presentation as well as for subsequent computations of participants’ walking speed. The average walking speed was calculated as the mean velocity over all walking trials per person and condition. To account for individual acceleration and deacceleration phases at the beginning and the end of each walk, the first two and the last two meter were discarded from the analysis. No task prioritization instruction was given.

### Data recordings and procedures

100 visual stimuli were presented for each eccentricity in each motor task condition (sitting, standing, walking). The resulting 1800 visual stimuli were distributed over 12 experimental blocks and performed in an individually alternating but counterbalanced order for both age groups. Before each recording, baseline walking speed was measured over a walking distance of 50 m in length. For three participants, walking speed recordings during the experimental manipulation were only available for three out of four blocks. Recording times including breaks were about 2 h in total. On average, 527 trials (*SD*: 24.8) from the sitting condition and 495 trials (*SD*: 55.2) from the walking condition entered the preprocessing pipeline.

### EEG recordings and preprocessing

EEG data were measured with a mobile system using actively amplified electrodes (MOVE, Brain Products GmbH, Gilching, Germany). Data from 64 channels, arranged according to the extended international 10%-system^18^, were recorded. Electrodes AF7 and AF8 were placed under the left and right eye, respectively, and later used to assess electroocular activity. Data preprocessing was performed with Matlab 2015a and Matlab 2020a (MATLAB, The MathWorks Inc., Natick, MA, USA), EEGLAB functions^19^ and custom scripts. Data were first highpass filtered (0.1  Hz) and lowpass filtered (100 Hz). Defect channels or channels with prominent artifacts were then rejected based on predefined rejection criteria (5 *SD* of the mean kurtosis value or 3*SD* from mean probability distribution of each single channel) and subsequently checked manually ($M= 4.8, SD=2.5$). Afterwards, all channels were re-referenced to a common average reference.

A copy of the resulting data set was created that was (1 $\mathrm {Hz}$) highpass filtered and visually inspected for extensive artifacts in the time domain (e.g. muscle artifacts). This data set was decomposed using adaptive mixture independent component analysis algorithm (AMICA^20^). The resulting independent component (IC) weights were mapped back onto the original (0.1 $\mathrm {Hz}$) highpass filtered set, which was subsequently filtered again with a 40 $\mathrm {Hz}$ lowpass filter. Rejected electrodes were replaced using spherical spline interpolation. The standard boundary element method (BEM) head model implemented in the EEGLAB Dipfit plugin was used to localize equivalent dipole models for each IC. Subsequently, only ICs that were classified by automated classification (ICLabel, Version 1.2)^21^ as ICs reflecting brain sources ($M= 17.13, \,SD= 4.30)$ were included into further analysis. As we expected pronounced non-neural artifacts in the mobile EEG recordings, a classification threshold of $>50 \%$ was chosen. The resulting data sets were segmented into epochs of 2.5 *s* length, starting 1000ms before visual stimulus onset. Epochs exceeding a threshold criterion of 80mV were discarded.

### Event-related spectral perturbation (ERSP)

For each IC of each participant, single-trial spectrograms were calculated with EEGLAB function newtimef using Morlet wavelets with 3 cycles for lowest frequency and a linear 0.5 increase in cycles resulting in up to 15 cycles for the highest frequencies. Spanning a frequency range of 4 - 40Hz, this resulted in 37 log-spaced frequencies. Furthermore, all trials were time-warped to equal stimulus-response intervals (relative to the stimulus onset time and to the mean response time over all participants and trials: 356ms after stimulus onset) using linear interpolation.

### Repetitive IC clustering

For group level comparison, all remaining ICs were first clustered based on their equivalent dipole location (weight =10) and their average ERSP (weight = 3). The final feature vector of the weighted measures was compressed to the first 10 dimensions using PCA. All ICs were assigned to 14 cluster based on their distance in the feature vector space. ICs that were 3 *SD* away in vector space from the cluster centroids were classified as outliers. From this initial clustering solution, the cluster centroid locations of a left and right centrolateral cluster near the central sulcus representing left and right motor cortex areas were extracted.

Subsequently, we used a repetitive clustering approach, as described in Gramann et al. (2018)^22^, to determine a reproducible cluster solution. Therefore, we repeated the clustering procedure 10,000 times and selected an optimized solution for a predefined region of interest. The repetitive clustering was performed twice, once with the left centrolateral centroid coordinates (talairach coordinates x: − 33, y: − 10, z: 49) and once with the right centrolateral centroids (talairach coordinates x: 38, y: − 7, z: 48) from the initial clustering. The final selection of the clustering solution was based on weighting the number of participants assigned to that cluster (weight = 3), the number of ICs per subject within that cluster (weight = − 2), the cluster spread (weight = − 1), the mean residual variance of all cluster ICs (weight = − 1), the cluster distance to the ROI (weight = − 1), and the Mahalonbian distance of the cluster to the median of all solutions (weight = − 1).

**Figure 1:**
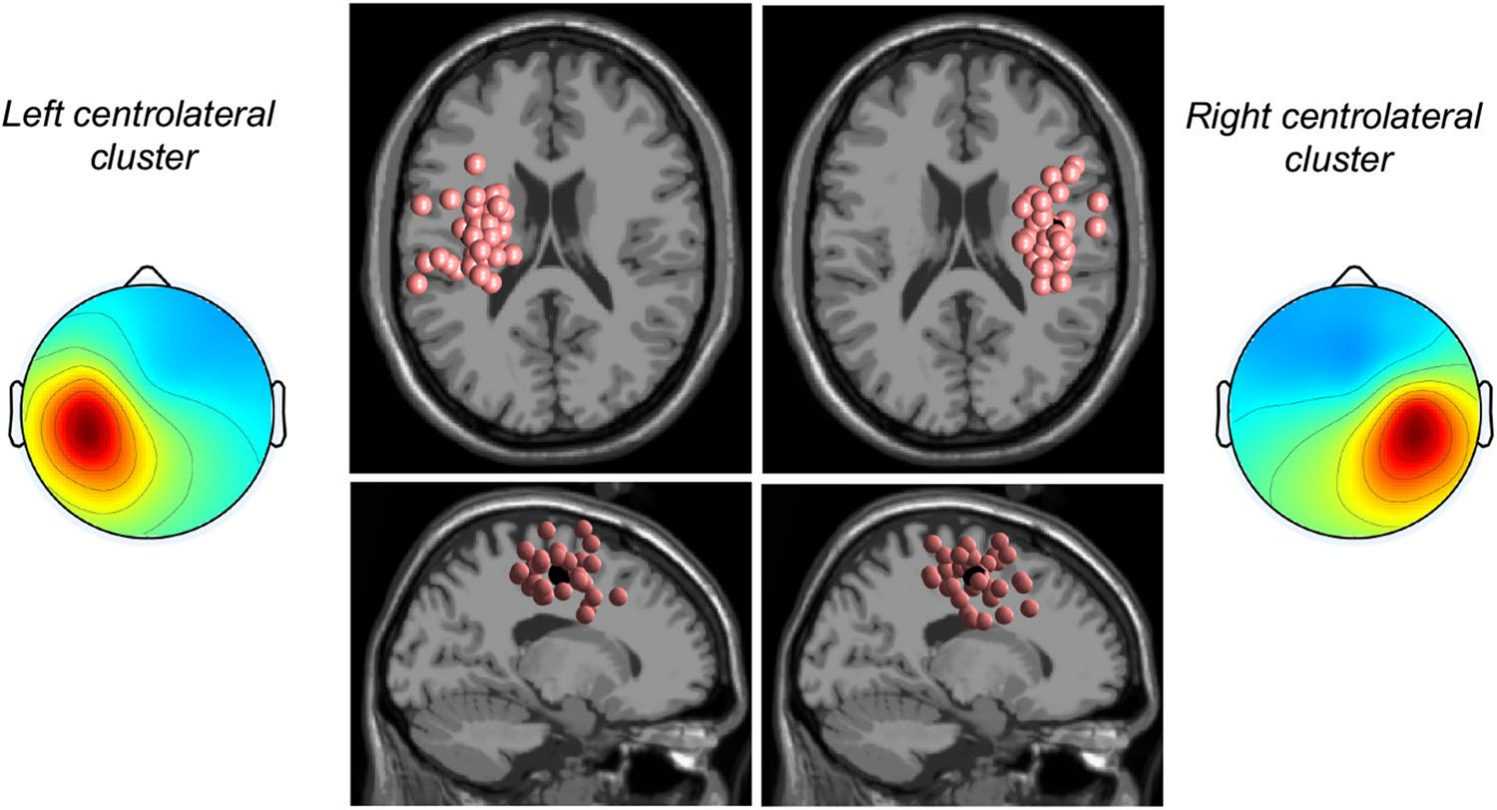
Middle columns: Equivalent dipole locations of the left and right centrolateral cluster ICs. Each individual IC is depicted by a smaller sphere (coral red) and the centroid of each cluster is represented by a larger sphere (black). Left and right: Scalp projections of each cluster mean.

From each of the two cluster solutions, the optimized cluster were extracted (see Fig. 1), with cluster centroids near the left and right central sulcus (talairach coordinates right: x: − 34, y: − 9, z: 49, talairach coordinates left: x: 39, y: − 7, z: 49). Based on the obtained scalp maps, the approximated source location of both clusters were in a reasonable range (based on the expected spatial accuracy of EEG dipole localization) of the assumed hand area of the sensorimotor cortex. 38 ICs were included in the right centrolateral cluster, with 23 ICs from 15 younger participants and 15 ICs from 13 older participants. The left centrolateral cluster consisted of 35 ICs with 18 ICs from 12 participants from the younger group and 17 ICs from 13 participants from the older group. There was no overlap in ICs between the left and right cluster solutions.

### Motor cluster power spectral density (PSD) and 1/f examination

Individual mean PSD from 0.1 to 40Hz were calculated for each motor cluster IC over all epochs of each motor task condition using fast Fourier transforms. Individual PSD values were parameterized as the power of the identified peaks within the alpha (8–12 Hz) and beta (16–30 Hz) frequency band ranges. For the assessment of potential systematic age-group or motor-task differences in the aperiodic proportion of the averaged PSD of each participant, we parameterized the aperiodic component using the python *FOOOF-Toolbox*^23^. The toolbox assumes that aperiodic 1/f activity can be separated from the periodic proportion (defined as power over and above broadband 1/f-activity) of an EEG power spectrum. In iterative steps, the aperiodic activity is fitted and removed from the power spectrum. Frequency peaks are then identified and fitted with a Gaussian kernel in the 1/f-corrected and thus flattened spectrum. Please see Donoghue et al. (2020)^23^ for details of the fitting procedure. For the current analysis, we fitted the aperiodic spectra without knees. For the statistical assessment of potential differences in the approximated aperiodic activity, the exponent and the offset of the 1/f-like broadband activity were extracted for each participant.

### Group-level ERSP analysis

All ICs included in the left centrolateral (n =35) and right centrolateral cluster (n=38) entered the ERSP analysis. On the IC level, TF data were first normalized by dividing all single trial data by the mean of the respective full epoch, as proposed by Grandchamp & Delorme (2011)^24^. Afterwards, all epochs were averaged per IC for each motor task condition. On the level of single participants, the TF data were averaged and decibel (dB) transformed over all ICs per participant and condition. Event-related desynchronization associated with the button press was assessed per condition and group as the proportional difference in power between the baseline window before response execution (− 300:− 100 ms relative to stimulus onset) and the power related to the button presses. The data extraction for the statistical analysis was guided by a data driven approach. We accounted for individual differences in the timing and peak frequency of alpha and beta band activity^25^ for each person in each condition. For this purpose, we first identified the maximal desynchronization in the alpha (8–12 Hz) and beta band (16–30 Hz) from the grand average ERSP over all participants and conditions. We then identified the time points and peak frequencies of each individual desynchronzation maxima for each band and in each condition in a 200 ms time window (+/− 100 ms) around the time point of the maximum alpha desynchronization (378 ms after stimulus onset) and beta desynchronization (562ms after stimulus onset) of the grand average. For the statistical analysis, we computed the average power in a time window of +/− 20 ms around the time point of the individual participant’s maximum suppression (window length = 41 ms) within a frequency range of +/− 1 of neighboring log-spaced frequencies for the alpha band and +/− 2 log-spaced frequencies for the broader beta band. The mean of the resulting center frequency distribution was 10.5 Hz (SD: 1.4 Hz) for the alpha band and 21.0 Hz (SD: 4.0 Hz) for the beta band and thus in accordance with the expected peak frequencies for movement-related desynchronization.

### Design and statistical analysis

#### Assessment of potential confounding factors

##### Subgroup analysis

Because not every participant showed one IC in each motor cluster (seven out of thirty participants), we first compared analysis of variance (ANOVA) results for the dependent variable “alpha suppression” and “beta suppression”, averaged over both motor clusters, for a 2 × 2 factorial mixed-measures design with the within factor age-group and the between factor motor condition for the subgroup that showed ICs in each of the two motor clusters (N = 23, n_old_ = 11, n_young_ = 12). Subsequently, to increase the number of participants for the statistical analyses, we included all participants with one IC in any of the motor clusters of interest, irrespective of whether the same participant showed an IC in the other motor cluster (i.e., the entire group; N = 30, n_old_ = 15, n_young_ = 15). As we did not find any difference in the effect patterns (despite slightly higher effect sizes in the subgroup), we decided to report results of the entire sample in this report. Complete results of the subgroup analysis can be found in the supplementary information (table S1).

##### Factor reduction

**Figure 2:**
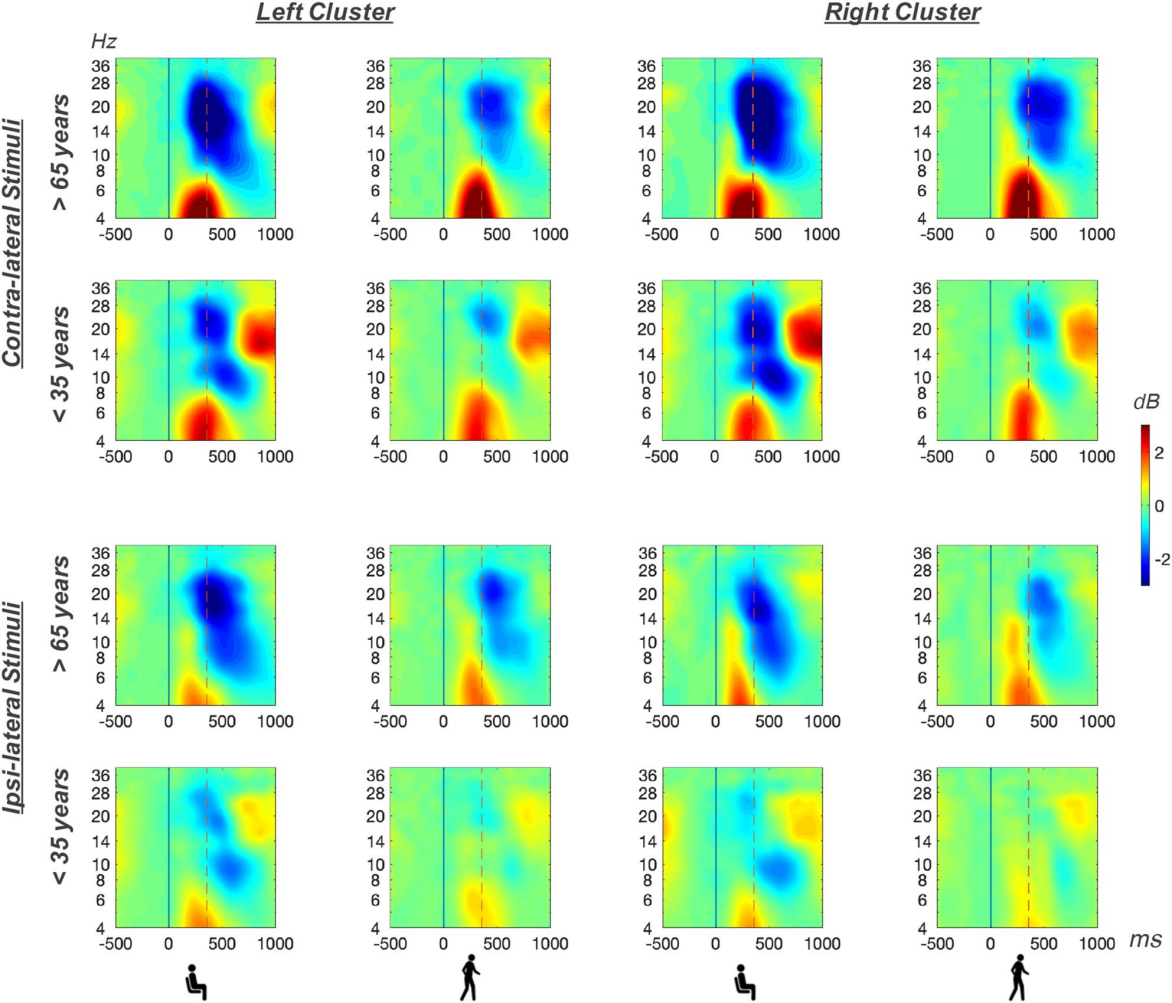
Mean ERSPs for the left centrolateral cluster (left two columns) and the right centrolateral cluster (right two columns) for contra-lateral presented stimuli (upper two rows) and ipsi-lateral presented stimuli (lower two rows), separately for older participants (fist and third row) and younger participants (second and fourth row) during sitting (first and third column) and walking (second and fourth column). ERSP trials were time-warped with respect to the stimulus onset (time point zero, solid line in black) and the mean button press response time (356ms after stimulus onset, dashed vertical line in red).

We further used participants with an IC in both cluster (N = 23) to analyze the potential confounding factors a) manual response side (left and right index finger button presses) and b) lateralization effects (ipsilateral and contralateral hemisphere relative to response side) on the intended age group and motor task comparisons. For this purpose, we ran a 2× (age group) 2× (motor task) 2× (response side) 2× (lateralization) ANOVA to check for interaction effects including the dependent variables beta and alpha suppression. For both frequency bands, a significant interaction of motor condition and hemisphere was obtained (see Fig. 2). As expected, higher desynchronization levels were found in the contralateral cluster relative to the response side as compared to the ipsilateral cluster. Furthermore, desynchronization patterns were more pronounced during sitting as compared to walking. However, no motor task specific confounding differences in hemispheric activation reached significance (all *p* values > 0.05). As no further interaction effects involving hemisphere or response side with age group and motor condition were observed, subsequent analyses focused on age and motor condition only, since lateralization effects were not in the scope of this study. The following analyses were thus performed on the average over both motor cluster. Complete test statistics of all main and interaction effects of this analysis are listed in the supplementary information (table S2).

### Statistical analysis

Differences in the overall PSD and in the modeled aperiodic component of the PSD as well as in button press-related dB power were assessed by 2 × 2 ANOVAs with the within factor motor task (sitting, walking) and the between factor age group (< 35 years, ≥ 70 years). T-Tests on differences in estimated marginal means were calculated for post-hoc comparisons. Resulting *p* values were FDR adjusted based on the number of comparisons. Since ERSP calculations always depend on the reference period (in this case − 300 to − 100 ms before the button press), an additional 2 × 2 ANOVA with the within factor motor task (sitting, walking) and the between factor age group (< 35 years, ≥ 70 years) was calculated for power values in this period. The main effect pattern matched the overall PSD pattern and we thus decided to only report and discuss the overall PSD values to avoid redundancy. Complete results can be found in the supplementary information (table S3).

For the assessment of relative changes from single to dual-task conditions, proportional dual-task costs were calculated as proposed by Lindenberger et al. (2000)^26^: [(Single Task Score - Dual Task Score)/Single Task Score] *100.

Distributions of the resulting ERSP dual-task cost data were found to be skewed and therefore transformed using ordered quantile normalization^27^ prior to the statistical analysis. The transformed values follow a normal distribution with highest positive values indicating highest dual-task costs (maximal observed reduction in desynchronization from sitting to walking) than positive values. Group differences in dual-task costs were assessed by Welch two-sample t-Tests.

For the analysis of linear associations between ERSP estimates, visual task performance, and walking speed, partial correlations were calculated for the whole sample using partial Spearman rank correlations, controlling for age, for absolute and dual-task cost measures. These measures were selected for the analysis of overall relationships in resource allocation processes for both parallel performed motor-tasks. In addition, confirmatory correlations were calculated to control for further potentially mediating associations between button-press related ERSP values and several visual task measures (response times, misses as well as P1 and P3 amplitudes). The latter analyses were primarily executed to uncover if desynchronization levels are potentially driven by motor task specific changes in processing on earlier perceptual or central stages. All statistical analyses were performed using R^28^.

## Results

An overview of all effects can be found in table 1.

**Table 1:** Overview of ANOVA main and interaction effects of the PSD and TF analysis.

Parameter	Age	Motor condition	Age × motor condition
*PSD alpha*	–	Sit > Walk	–
*PSD beta*	Old > Young	–	–
*Modeled PSD aperiodic exponent*	–	–	–
*Modeled PSD aperiodic offset*	–	Sit < Walk	–
*ERSP alpha*	–	Sit < Walk	–
*ERSP beta*	Old < Young	Sit < Walk	–

### PSD and 1/f activity

PSD modulations within the alpha and beta band as well as the modeled aperiodic component and offset were statistically assessed to analyze the overall frequency pattern of each group in each motor condition. For PSD values within the range of the alpha band a significant effect of motor task, $F(1,28)=8.37, \, p=0.007, \,\eta ^{2}_{g}= 0.04$, was revealed with higher power during sitting ($M= -1.57\,SD= 0.40$) as compared to walking ($M= -1.70 \,SD= 0.29$). No age group, $F(1,28)=0.02, \, p=0.891, \,\eta ^{2}_{g}< 0.01$, or interaction effect, $F(1,28)=0.01, \, p=0.907, \,\eta ^{2}_{g}< 0.01$, on alpha power was found. For the beta band, a significant effect of age group, $F(1,28)=4.65, \, p=0.040, \,\eta ^{2}_{g}= 0.11$, was found with higher beta power in the older group ($M= -1.83 \,SD= 0.22$) as compared to the younger group ($M= -2.00 \,SD= 0.29$). No further effects were revealed for the PSD values in the beta band (motor task: $F(1,28)=0.40, \, p=0.532, \,\eta ^{2}_{g}< 0.01$, age group × motor task: $F(1,28)=1.26, \, p=0.272, \,\eta ^{2}_{g}= 0.01$).

**Figure 3:**
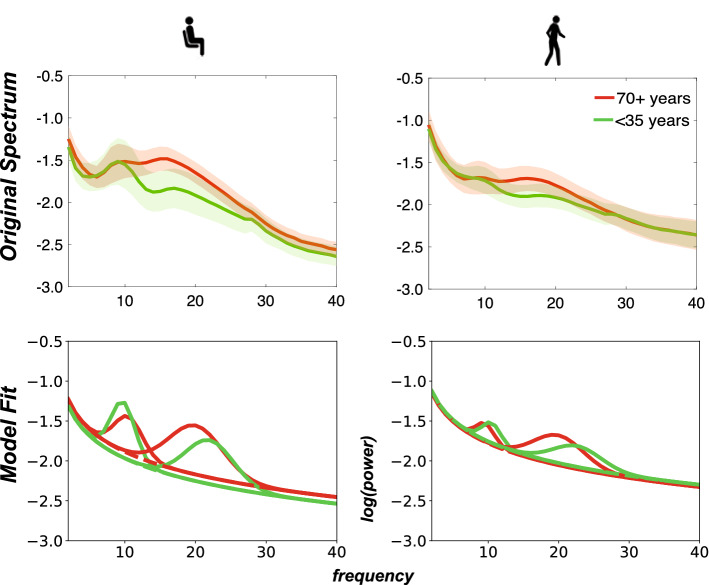
Upper row: Mean PSD traces over both centrolateral clusters from 4 to 40 Hz during sitting (left) and walking (right). A 95% confidence interval for each group is indicated by the surrounding envelope in the corresponding color. Lower row: Mean model fit of the spectra obtained by the FOOOF-algorithm^23^.The dashed line is the fit of the aperiodic component. Mean traces for older participants are red and for younger participants they are green in all four plots.

For the modeled aperiodic component of the PSD, only a significant effect of motor task on the offset was found, $F(1,28)=7.41, \, p=0.011, \,\eta ^{2}_{g}= 0.03$ (see Fig. 3). The offset significantly increased from sitting ($M= -0.98 \,SD= 0.35$) to walking ($M= -0.86 \,SD= 0.35$). No significant age group, $F(1,28)=0.07, \, p=0.799, \,\eta ^{2}_{g}< 0.01$), or interaction effect, $F(1,28)=1.29, \, p=0.266, \,\eta ^{2}_{g}= 0.01$, was found for the offset of the aperiodic component. Furthermore, no effects were observed for the analysis of difference in the exponent of the aperiodic signal of the power spectrum between 2 and 40 Hz (age group: $F(1,28)<0.01, \, p=0.978, \,\eta ^{2}_{g}< 0.01$, motor task: $F(1,28)=1.10, \, p=0.302, \,\eta ^{2}_{g}= 0.01$, age group × motor task: $F(1,28)=0.01, \, p=0.925, \,\eta ^{2}_{g}< 0.01$).

### ERSPs

**Figure 4:**
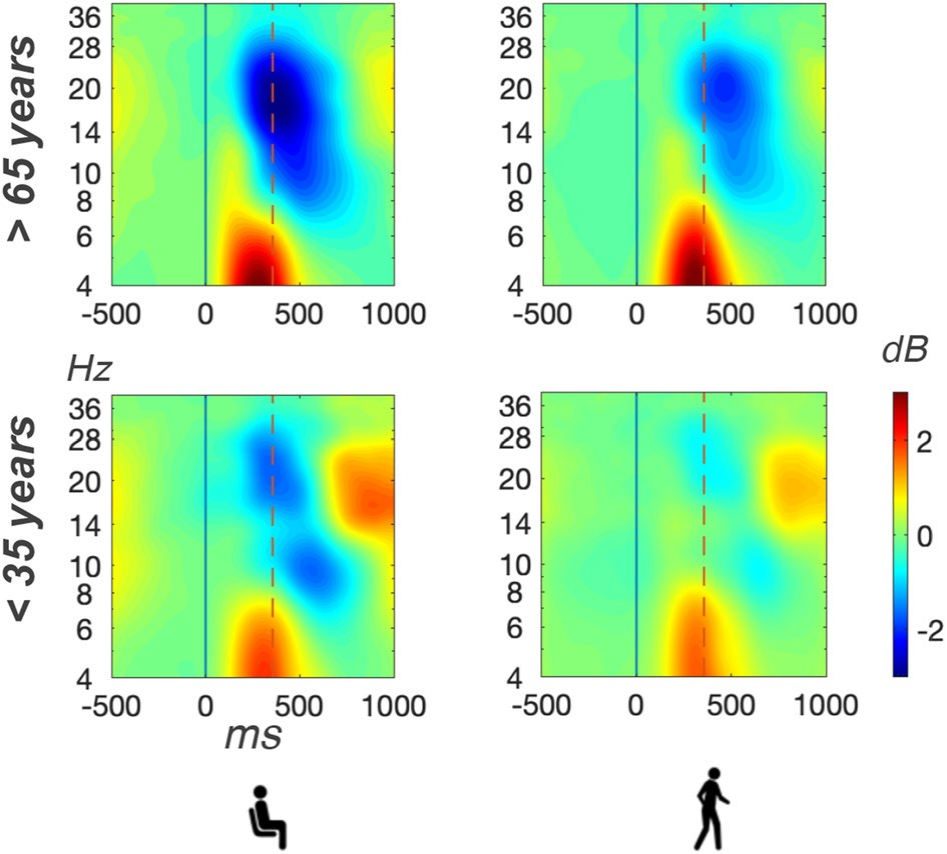
Mean ERSPs over both centrolateral clusters for older participants (upper row) and younger participants (lower row) during sitting (left column) and walking (right column). ERSP trials were time-warped with respect to the stimulus onset (time point zero, solid line in black) and the mean button press response time (356 ms after stimulus onset, dashed vertical line in red).

**Figure 5:**
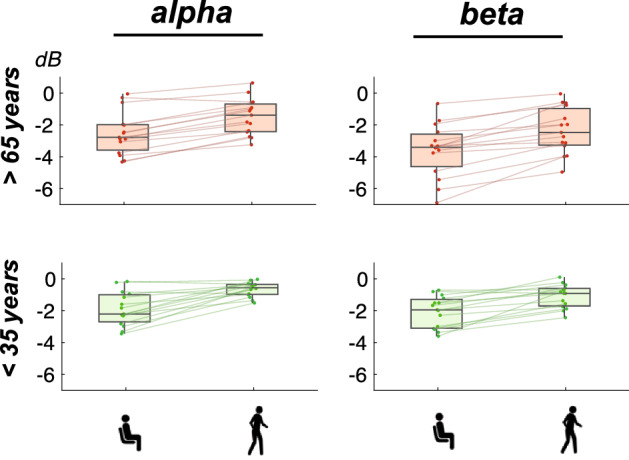
Distribution of button-press related alpha desynchronization values (left column) and beta desynchronization values (right column) in dB in older participants (upper row, in red) and in younger participants (lower row, in green). Scatter points indicate individual mean values for each participant. Within each plot, the means of each participants during sitting and walking are connected by lines.

ERSP changes within the alpha and beta band were calculated for the analysis of button-press related frequency modulations within each group and motor condition. For alpha suppression accompanying the button presses, we found a significant main effect of motor task, $F(1,28)=69.00, \, p<0.001, \,\eta ^{2}_{g}= 0.24$ (see Figs. 4 and 5). Higher desynchronization values were found during sitting ($M= -2.20, \,SD= 1.23$) as compared to walking ($M= -1.07, \,SD= 0.94$). No significant age group difference was found for the alpha frequency range, $F(1,18)=4.06, \, p=0.054, \,\eta ^{2}_{g}= 0.11$, nor was there an interaction of the two factors $F(1,28)=0.54, \, p=0.467, \,\eta ^{2}_{g}< 0.01$.

The analysis of button press-related beta suppression revealed significant main effects for age group, $F(1,28)=10.13, \, p=0.004, \,\eta ^{2}_{g}= 0.25$, and motor task, $F(1,28)=65.78, \, p<0.001, \,\eta ^{2}_{g}= 0.17$ without interaction of the factors, $F(1,28)=0.90, \, p=0.352, \,\eta ^{2}_{g}< 0.01$. The beta band desynchronization relative to the baseline was more pronounced in older participants ($M= -2.97, \,SD= 1.65$) as compared to younger participants ($M= -1.57, \,SD= 0.98$). In addition, higher beta desynchronization values were found during sitting ($M= -2.83, \,SD= 1.55$) as compared to walking ($M= -1.71, \,SD= 1.29$).

### ERSP dual-task costs

Relative changes in button-press related alpha and beta suppression were analyzed as dual-task cost for each group and motor condition. Significant smaller proportional reductions in button press-related desynchronzations from sitting to walking were found in older participants (alpha: $M= -2.00, \,SD= 1.33$; beta: $M= -2.97, \,SD= 1.65$) compared to younger participants (alpha: $M= -1.28, \,SD= 1.01$; beta: $M= -1.57, \,SD= 0.98$) for the alpha band, $t(28) = -2.36, p=0.022$, and the beta band, $t(28) = -3.97, p<0.001$.

### Correlations

Partial correlations were calculated for the evaluation of linear associations between walking task performance (operationalized as walking speed) and resource allocation measures for the parallel manual motor task (operationalized as button-press related ERSP modulations). In order to take associations between relative changes into account, dual-task costs were also included in the analysis for these measures. While alpha and beta suppression were found to be uncorrelated to walking speed, dual-task costs in power were negatively correlated with dual-task costs in walking speed (alpha: $r(27)=-0.43, \,p=0.020$, beta: $r(27)=-0.50, \,p=0.006$). Greater reductions in alpha and beta desynchronization from sitting to walking were associated with smaller walking speed decrements. Results of the correlational analysis of the relationship of these measures with button press related activity revealed no significant association between any behavioral measure (misses, response time) or ERP feature (P1 and P3 amplitudes) reported in previous report^2^ and button press-related changes in the alpha and beta band (see supplementary information, table S4 and table S5). For the corresponding statistics and discussion on the main effects, the reader is redirected to original report.

## Discussion

We tested dual-task performance in younger and older participants within a scenario that resembled an everyday situation - the reaction to visual cues (e.g. upcoming cars) during locomotion (while approaching a street). Like in real-life situations, we did not instruct our participants to prioritize one task over the other. Thus, we were able to analyze age-related differences in the amount of resources allocated to each task. We revealed age-group and motor task specific effects for button press-related oscillatory activity. In addition, we observed an age-dependent reduction in dual-task related oscillatory modulation from sitting to walking.

### Increased beta desynchronization in older participants

In line with the current literature, we found a general increase in motor task-related desynchronization in older compared to younger participants that was specific for the beta band^8^. Based on the absence of an age-related effect for the modeled aperiodic offset of the PSD and on the assumption that the characteristics of the aperiodic components of the power spectrum are stable within trials, the results of the presented analyses of the TF activity cannot be explained by age-group differences in the aperiodic component.

However, the overall PSD values in beta range and thus the beta power in the reference period for the ERSPs were increased in older compared to younger adults. The relatively higher button-press related beta desynchronisation in older participants could therefore be necessary to compensate for higher baseline activation level. Heinrichs-Graham and Wilson (2016)^29^, for example, argue that higher baseline beta power in older participants potentially lead to higher beta desynchronisation, as a certain threshold of beta desynchronssation must be reached to execute a movement.

On a functional level, the amount of beta suppression was neither linked to overt visual task performance nor to walking speed in the two age groups. Without a link to behavior or additional information about neural inefficiency in the older age group, the obtained general local upregulation in beta desynchrony in older participants during button presses cannot be interpreted explicitly as compensatory or as inefficient^30^. For further functional interpretation, we further investigated the time course of alpha and beta band desynchronization accompanying correct button presses during sitting and during the more complex motor task of walking.

### Reduced alpha and beta desynchronization during walking

Alpha and beta desynchronization accompanying button presses where more pronounced during the seated condition compared to the walking condition. Furthermore, no direct relation between power modulation and visual task performance like response time or accuracy was observed. Despite missing timing information of the button press-related desynchronization due to our warping approach, this result strengthens the assumption that decrements in visual task performance during walking are reflected in deficient early visual processing rather than later processing steps related to motor preparation and output^2^. Furthermore, the amount of desynchronization was not associated with amplitude measures of early visual (P1) or central (P3) information processing, ruling out that increased desynchronization in our study might be primarily driven by a degradation of perceptual or central information processing stages. As we only recorded response times and accuracy, we are not able to draw conclusions about other characteristics of the button press response (e.g. strength or duration) in relation to alpha and beta suppression. However, previous studies reported beta desynchronization as rather insensitive to difference in movement type (e.g. slow vs. rapid finger movements, for a review see^31^). Furthermore, it has to be noted that the overall PSD decreased in the alpha frequency range from sitting to walking. Accordingly, the motor task of walking might already lead to a certain decrease of alpha power and thus contribute to the less pronounced button-press related desynchronization in the alpha range. This effect is possibly also reinforced by the increased broadband offset of the modeled aperiodic portion of the PSD during walking. However, a decrease in beta desynchronization from sitting to walking was observed with no differences in the overall PSD between these conditions. Based on this observation, the existence of an absolute threshold of beta desynchronsation for movement execution is highly unlikely. Despite a significant reduction from sitting to walking, button press-related desynchronization patterns in both conditions nonetheless reflected activity accompanying correct presses. Based on our result, we thus hypothesize that our participants potentially invested more resources than necessary, reflected in increased desynchronization, into the execution of a successful button press during the less complex seated task. This could be interpreted as a conservative or safety strategy in situations were more resources than needed are at disposal. During walking, however, this strategy has to be revised and resources must be redrawn from the button press task as they are needed for the parallel motor task. This interpretation is in line with Wicken’s (2002)^32^ limited resource theory, as both tasks draw on a shared pool of motor response resources. For a more detailed understanding of age-group differences in resource allocation to different motor tasks, we looked at relative power changes in terms of dual-task costs.

### Reductions in alpha and beta desynchronization during walking are inversely related to walking speed

Based on dual-task cost calculations, we analyzed the relative amount of resources that were drawn away from the motor response aspect of the visual task and thus potentially available for a reassignment to the walking task. The analysis of the modulation of alpha and beta desynchronization provided two important insights. First, the reduction in alpha and beta desynchronization from sitting to walking was significantly smaller in older compared to younger participants. Thus, younger participants revealed higher reductions in button press-related power suppression than older participants. Second, the strength of the reduction within these bands was inversely correlated with walking speed. In other words, participants who revealed the least reduction in beta and alpha desynchronization from sitting to walking showed the highest reduction in walking speed from baseline to dual-task walking. This reflects a resource allocation strategy that fosters resources to be allocated to the button-presses rather than the walking task.

Given the missing association between visual task performance and the amount of desynchronization in both groups and motor conditions, we can only speculate on an underlying functionality of reduced modulations for visual task performance in older participants. Either older participants needed increased desynchronization to sustain performance during the more complex task of walking, or they were less able to allocate resource flexible between both tasks. The latter would point to differences in resource allocation abilities rather than to a mere resource capacity problem. However, further research is needed to answer if more pronounced resources allocation to the button press is functional or rather inefficient for visual task performance. In contrast, we were able to show a direct relationship of the proportional reduction in button press-related sensorimotor desynchronization and walking task performance. The less participants were able to withdraw resources from the execution of the button-press, the slower they walked. In addition to the significantly less pronounced reductions in alpha and beta desynchronization from sitting to walking, only older participants reduced their walking speed significantly from baseline to dual-task walking, as previously reported^2^. We thus hypothesize that older participants have less resources available than younger participants for the walking tasks as relatively more resources are being withhold for response execution of the button press. As walking performance was directly linked to changes in brain dynamics underlying response execution in the concurrent task during dual-task walking, we propose modulations in movement-related sensorimotor desynchronization as a neurophysiological marker of CMI in two concurrent performed motor tasks.

### Limitations

Several limitations of the present work have to be mentioned. First, we did not record motion tracking data for a complex gait cycle analysis. As a result, we were unable to assess potential individual and dynamic within gait cycle coordination between both tasks as recently proposed by Smeeton et al. (2021)^33^. In addition, detailed information about gaze behavior or the strength and duration of each motor response would have covered a wider range of brain-behavior relationships in dual-task walking. Finally, future studies should aim at larger and broader age group sample sizes in order to carry out meaningful within-group analysis.

### Conclusion and outlook

In summary, we have found age-group and motor task specific differences in button press-related sensorimotor activity. Importantly, the proportional reduction in alpha and beta desynchronization from sitting to walking was less pronounced in older participants and linked to the adaption of gait speed in dual-task walking. We thus provided first neurophysiological evidence for age group differences in allocations strategies in motor resource conflicts during dual-task walking. Based on our results, future studies with corresponding sample sizes should explore age-group specific associations of power modulations and behavior in dual task scenarios using analysis of whole-brain network dynamics. For a holistic understanding of modulation of behavior in motion, connectivity estimation between motor and e.g. prefrontal areas might be crucial. By adopting this approach, we expect to build a neuroscientific basis for the evaluation of training programs aiming at attenuating motor dual-task costs, and most important, to gather a profound understanding of the processes that should be trained.

## Supplementary Information

Supplementary Tables.

## Data Availability

The datasets analysed during the current study are available from the corresponding author on reasonable request.
