# Supplementary material for: EEG beta-modulations reflect age-specific motor resource allocation during dual-task walking
Source: Sci Rep. 2021 Aug 9;11:16110. doi: 10.1038/s41598-021-94874-2 (PMC8352863; doi:10.1038/s41598-021-94874-2)
Supplement: Supplementary file 1 — Supplementary Tables. [file 41598_2021_94874_MOESM1_ESM.pdf]

Supplemental material for the manuscript: *EEG Beta-Modulations reflect Age-Specific Motor Resource Allocation during Dual-Task Walking*

Janna Protzak[1*] **and Klaus Gramann**[2]

[1]Junior research group FANS (Pedestrian Assistance System for Older Road User), Technische Universitaet Berlin, 10587 Berlin, Germany

[2]Biological Psychology and Neuroergonomics, Technische Universitaet Berlin, 10623 Berlin, Germany

[*]janna.protzak@tu-berlin.de

Table 1. Overview of ANOVA main and interaction effects of the subgroup analysis.

Effect	alpha		beta	
Age	*F(1,21)=4.05*	, $p = 0.057, \eta_g^2 = 0.15$	*F(1,21)=13.02*	$p = 0.002, \eta_g^2 = 0.36$
Motor Task	*F(1,21)=68.75*	, $p < 0.001, \eta_g^2 = 0.28$	*F(1,21)=49.98*	$p < 0.001, \eta_g^2 = 0.21$
Age x Motor Task	*F(1,21)=1.34*	, $p = 0.260, \eta_g^2 = 0.01$	*F(1,21)=0.45*	$p = 0.509, \eta_g^2 < 0.01$

Table 2. Overview of ANOVA main and interaction effects of the full design analysis, A= Age, MT = Motor Task, PS = Presentation Side, CH = Cluster Hemisphere.

**Effect**	**alpha**	**beta**
Age	$F(1,21) = 3.13, p = 0.091, \eta_g^2 = 0.08$	$F(1,21) = 10.51, p = 0.004, \eta_g^2 = 0.23$
MT	$F(1,21) = 71.11, p < 0.001, \eta_g^2 = 0.20$	$F(1,21) = 66.37, p < 0.001, \eta_g^2 = 0.17$
CH	$F(1,21) = 23.91, p < 0.001, \eta_g^2 = 0.04$	$F(1,21) = 47.40, p < 0.001, \eta_g^2 = 0.14$
PS	$F(1,21) = 4.21, p = 0.053, \eta_g^2 < 0.01$	$F(1,21) = 7.96, p = 0.010, \eta_g^2 = 0.14$
A x MT	$F(1,21) = 1.78, p = 0.196, \eta_g^2 = 0.01$	$F(1,21) = 0.78, p = 0.389, \eta_g^2 < 0.01$
A x CH	$F(1,21) = 1.70, p = 0.207, \eta_g^2 = 0.01$	$F(1,21) = 1.47, p = 0.239, \eta_g^2 < 0.01$
A x PS	$F(1,21) = 0.07, p = 0.796, \eta_g^2 < 0.01$	$F(1,21) = 0.44, p = 0.513, \eta_g^2 = 0.01$
MT x CH	$F(1,21) = 9.11, p = 0.007, \eta_g^2 = 0.01$	$F(1,21) = 21.30, p < 0.001, \eta_g^2 = 0.01$
MT x PS	$F(1,21) = 1.88, p = 0.185, \eta_g^2 < 0.01$	$F(1,21) = 1.52, p = 0.231, \eta_g^2 < 0.01$
CH x PS	$F(1,21) = 0.67, p = 0.422, \eta_g^2 = 0.01$	$F(1,21) = 0.10, p = 0.760, \eta_g^2 < 0.01$
Ax MT x CH	$F(1,21) = 0.53, p = 0.476, \eta_g^2 < 0.01$	$F(1,21) = 0.71, p = 0.409, \eta_g^2 < 0.01$
Ax MT x PS	$F(1,21) = 0.65, p = 0.430, \eta_g^2 < 0.01$	$F(1,21) = 0.02, p = 0.885, \eta_g^2 < 0.01$
Ax CH x PS	$F(1,21) = 0.18, p = 0.672, \eta_g^2 < 0.01$	$F(1,21) = 0.69, p = 0.415, \eta_g^2 = 0.01$
MT x CH x PS	$F(1,21) = 0.12, p = 0.732, \eta_g^2 < 0.01$	$F(1,21) = 0.42, p = 0.526, \eta_g^2 < 0.01$
A x MT x CH x PS	$F(1,21) = 0.26, p = 0.618, \eta_g^2 < 0.01$	$F(1,21) = 0.50, p = 0.485, \eta_g^2 < 0.01$

Table 3. Overview of ANOVA main and interaction effects of the ERSP reference period analysis with post-hoc t-test results. Please note that post-hoc test for the interaction effect on beta power only confirmed the significant main effect of age group.O = old, Y = young, S = sit, W = walk

Parameter	Age	Motor Condition	Age x Motor Task
alpha	*F*(1,28)= 2.44, *p* = 0.130<br>$\eta_g^2 = 0.07$	*F*(1,28)= 24.92, *p* < 0.001<br>$\eta_g^2 = 0.13$<br>S>W: *t*(28)= 4.99,*p* < 0.001	*F*(1,28)= 0.79, *p* = 0.383<br>$\eta_g^2 = 0.01$
beta	*F*(1,28)= 18.51, *p* < 0.001<br>$\eta_g^2 = 0.34$<br>O>Y: *t*(28)= 4.30,*p* < 0.001	*F*(1,28)= 0.57, *p* = 0.457<br>$\eta_g^2 = 0.01$	*F*(1,28)= 4.57, *p* = 0.041<br>$\eta_g^2 = 0.04$<br>O: S-W: *t*(28)= 2.05,*p* = 0.067<br>Y: S-W: *t*(28)=−0.98,*p* = 0.336<br>S: O>Y: *t*(28)= 3.92,*p* = 0.001<br>W: O>Y: *t*(28)= 3.86,*p* = 0.001

Table 4. Partial spearman rank correlation results controlled for age, absolute values.

		Sit	Walk
alpha	Response Time	*r*(27)=−0.150, *p* = 0.436	*r*(27)=−0.014, *p* = 0.941
	Misses	*r*(27)= 0.275, *p* = 0.149	*r*(27)=−0.169, *p* = 0.380
	P1 Amplitude	*r*(27)=−0.047, *p* = 0.810	*r*(27)=−0.049, *p* = 0.802
	P3 Amplitude	*r*(27)=−0.077, *p* = 0.692	*r*(27)=−0.127, *p* = 0.513
beta	Response Time	*r*(27)=−0.016, *p* = 0.934	*r*(27)=−0.112, *p* = 0.563
	Misses	*r*(27)=−0.057, *p* = 0.770	*r*(27)=−0.162, *p* = 0.400
	P1 Amplitude	*r*(27)=−0.072, *p* = 0.712	*r*(27)= 0.257, *p* = 0.178
	P3 Amplitude	*r*(27)= 0.011, *p* = 0.954	*r*(27)=−0.101, *p* = 0.599

Table 5. Partial spearman rank correlation results controlled for age, dual-task costs.

	alpha DTC	beta DTC
Response Time DTC	*r*(27)= 0.025, *p* = 0.900	*r*(27)=−0.074, *p* = 0.702
Accuracy DTC	*r*(27)= 0.015, *p* = 0.938	*r*(27)=−0.262, *p* = 0.170
P1 Amplitude DTC	*r*(27)=−0.110, *p* = 0.569	*r*(27)= 0.077, *p* = 0.693
P3 Amplitude DTC	*r*(27)= 0.211, *p* = 0.273	*r*(27)= 0.116, *p* = 0.548
